# Giant magneto-optical responses in magnetic Weyl semimetal Co_3_Sn_2_S_2_

**DOI:** 10.1038/s41467-020-18470-0

**Published:** 2020-09-15

**Authors:** Y. Okamura, S. Minami, Y. Kato, Y. Fujishiro, Y. Kaneko, J. Ikeda, J. Muramoto, R. Kaneko, K. Ueda, V. Kocsis, N. Kanazawa, Y. Taguchi, T. Koretsune, K. Fujiwara, A. Tsukazaki, R. Arita, Y. Tokura, Y. Takahashi

**Affiliations:** 1 Department of Applied Physics and Quantum Phase Electronics Center, University of Tokyo, Tokyo, 113-8656 Japan; 2 Nanomaterials Research Institute, Kanazawa University, Ishikawa, 920-1192 Japan; 3 RIKEN Center for Emergent Matter Science (CEMS), Wako, 351-0198 Japan; 4 Institute for Materials Research, Tohoku University, Sendai, 980-8577 Japan; 5 Deparment of Physics, Tohoku University, Sendai, 980-8578 Japan; 6 Tokyo College, University of Tokyo, Tokyo, 113-8656 Japan

**Keywords:** Magneto-optics, Terahertz optics, Topological insulators

## Abstract

The Weyl semimetal (WSM), which hosts pairs of Weyl points and accompanying Berry curvature in momentum space near Fermi level, is expected to exhibit novel electromagnetic phenomena. Although the large optical/electronic responses such as nonlinear optical effects and intrinsic anomalous Hall effect (AHE) have recently been demonstrated indeed, the conclusive evidence for their topological origins has remained elusive. Here, we report the gigantic magneto-optical (MO) response arising from the topological electronic structure with intense Berry curvature in magnetic WSM Co_3_Sn_2_S_2_. The low-energy MO spectroscopy and the first-principles calculation reveal that the interband transitions on the nodal rings connected to the Weyl points show the resonance of the optical Hall conductivity and give rise to the giant intrinsic AHE in dc limit. The terahertz Faraday and infrared Kerr rotations are found to be remarkably enhanced by these resonances with topological electronic structures, demonstrating the novel low-energy optical response inherent to the magnetic WSM.

## Abstract (web summary)

The evidence of topological origin for the recently observed anomalous Hall effect remains elusive. Here, the authors report that the resonance of the optical Hall conductivity resulted from topological electronic structure gives rise to the large intrinsic anomalous Hall effect in the magnetic Weyl semimetal Co_3_Sn_2_S_2_.

## Introduction

The materials hosting the topological electronic band structure potentially exhibit exotic or enhanced electromagnetic responses^1–8^. The intrinsic anomalous Hall effect (AHE), which essentially differs from the scattering-process mediated extrinsic AHE, is a representative example of the topological transport phenomena, where the Berry curvature arising from the electronic band topology plays a decisive role for the Hall conductivity^9^. The intrinsic AH conductivity and AH angle are particularly enhanced by tuning the Fermi level to the (anti-)crossing point in the electronic band structure with Berry curvature^10^. Accordingly, the topological materials with nontrivial crossing points near their Fermi levels are attracting much attention as the ideal platforms for exploring the large AHE, accelerating the extensive material research^11–21^. One important advance is the recent discovery of the Weyl semimetal (WSM) possessing pairs of nondegenerate crossing points, i.e., Weyl points^1,14–17,20^. The pairs of Weyl points are characterized by the opposite chiralities and act as the monopole and anti-monopole of the emergent magnetic field in momentum space. The large AH conductivity and AH angle exceeding even 1000 Ω^−1^ cm^−1^ and 10%, respectively, are indeed reported for prospective WSMs^7,8,19,21^. However, because of the lack of the conclusive experimental proof, the role of the topological electronic structure for the large AHE remains elusive.

**Fig. 1:**
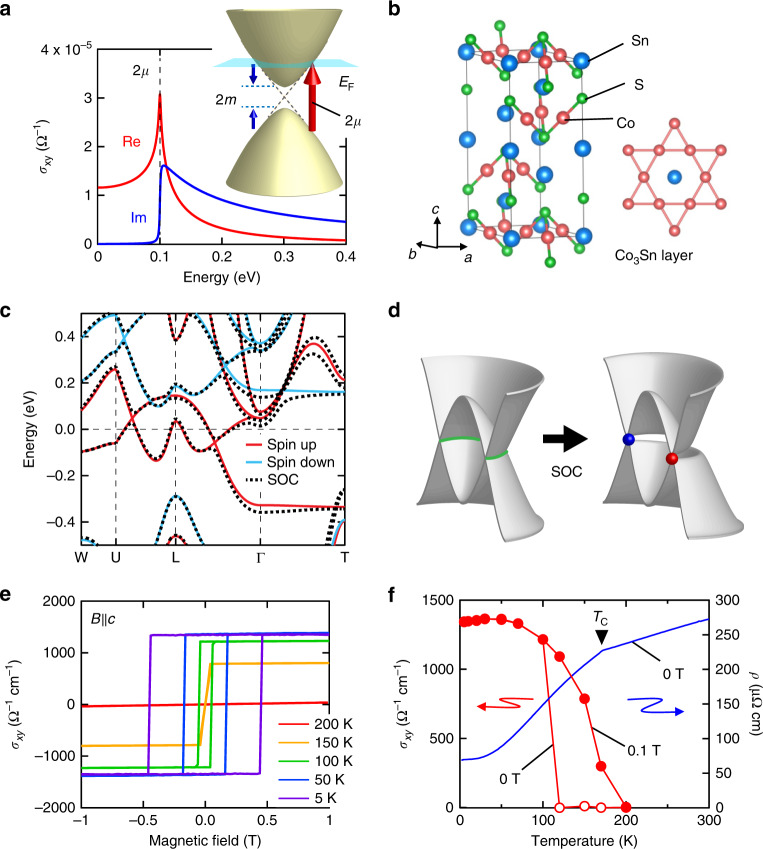
Electronic structure and anomalous Hall effect (AHE) of magnetic Weyl semimetal Co_3_Sn_2_S_2_. **a** Optical Hall conductivity *σ*_*xy*_(*ω*) calculated for a single anti-crossing point in the two-dimensional electronic structure with mass gap *m* = 0.03 eV and chemical potential μ = 0.05 eV. The red and blue curves are the real and imaginary parts of *σ*_*xy*_(*ω*), respectively. The inset shows the schematics of the band structure assumed in this calculation. **b** The crystal structure of Co_3_Sn_2_S_2_ and kagome network within Co_3_Sn layer. **c** Band structure obtained from the first-principles calculation. The red and blue lines represent the spin-up and spin-down bands, respectively, without spin–orbit coupling. The dotted line represents the band structure with the spin–orbit coupling. **d** Schematic illustrations of the simplified band structures: nodal ring structure (left) open the gaps with the anti-crossing nodal line connected to the Weyl points (right) due to the spin–orbit coupling. **e** Magnetic-field dependence of the Hall conductivity for each temperature when the magnetic field is applied parallel to the *c* axis for the bulk single crystal. **f** Temperature dependence of Hall conductivity at 0 T (red open circles) and at 0.1 T (red filled circles), and resistivity at 0 T (blue line) for the bulk single crystal.

The intrinsic AHE has been considered to exhibit the prominent feature in the optical Hall conductivity spectra *σ*_*xy*_(*ω*); the interband optical transition on the topological electronic structure with the Berry curvature gives rise to the resonant structure in *σ*_*xy*_(*ω*)^22–26^. We introduce an archetypal theoretical model for understanding the optical Hall conductivity spectra owing to the intrinsic AHE (Fig. 1a)^23^; the two-dimensional electronic structure has a single anti-crossing point with the mass gap 2 m, and the chemical potential μ is defined as the energy distance from the Weyl point. The vertical transition near the Weyl point causes the resonance peak at the energy of 2 μ in *σ*_*xy*_(*ω*), which corresponds to the lowest energy of the optical transition (Fig. 1a, inset; see also Supplementary Note 1). Im *σ*_*xy*_(*ω*) and Re *σ*_*xx*_(*ω*) (Supplementary Fig. 1) show the step function-like spectra at 2 μ^9,24^*,* indicating the dissipative response above the interband transition threshold. On the other hand, Re *σ*_*xy*_(*ω*) and Im *σ*_*xx*_(*ω*) represent the dissipationless responses, both of which show the sharp peaks and take finite value even below 2 μ. At *ω* = 0, the lower-lying tail of the resonance in Re *σ*_*xy*_(*ω*) equals to the Hall conductivity obtained by the DC transport measurement, so that the resonance intensity is responsible for the magnitude of AH conductivity. This spectral feature of the intrinsic mechanism is clearly distinguished from that of the extrinsic ones dominated by the intraband scattering. Therefore, the optical Hall conductivity spectra can provide the fingerprint of the intrinsic AHE.

Another intriguing aspect is the possibly giant magneto-optical (MO) Faraday/Kerr effect, i.e., the rotation of the light-polarization of the transmitted/reflected light for the magnetic media^27,28^, resulting from the enhanced Hall conductivity on the optical transition near the Weyl point; the magnitude of these MO effects is scaled by the optical Hall conductivity. This novel optical property inherent to the magnetic WSM remains to be explored.

In this work, we study the intrinsic AHE in terms of the MO response in the recently discovered magnetic WSM Co_3_Sn_2_S_2_ by using terahertz Faraday/infrared Kerr spectroscopy on the thin film/bulk single crystal and first-principles calculation. The thin film allows us to measure the optical response of the metallic compound around the terahertz region with high accuracy, where the low-energy limit of conduction electron dynamics can be examined. The observed low-energy resonance structure in the optical Hall conductivity is consistent with the DC AHE and the theoretical calculation, manifesting the intrinsic AHE arising from the topological electronic band structure. The MO Faraday and Kerr effects are largely enhanced by the interband transition on the topological electronic structures near the Fermi level, exemplifying the novel optical functionality of the topological materials.

## Results

### Magnetic Weyl semimetal

Co_3_Sn_2_S_2_, a Co-based shandite material, is a ferromagnetic metal with the Curie temperature *T*_C_ of ~175 K^7,8,20,21,29–31^. The crystal structure has a hexagonal lattice belonging to the space group *R*$\bar 3$*m* with forming a kagome network of Co atoms within the Co_3_Sn layer (Fig. 1b). Figure 1c reproduces the band structure of Co_3_Sn_2_S_2_ calculated by using the density functional theory (DFT) without/with the spin–orbit interaction, in accord with the literature^7,8^. In the case without the spin–orbit interaction, the spin-up band possesses the linear band crossings along the Γ-L and L-U directions above and beneath the Fermi level. Those crossing points are connected in three-dimensional reciprocal space, forming the nodal ring structure protected by a mirror symmetry of the crystal structure (Fig. 1d, left). When the spin–orbit interaction is taken into account, this nodal ring structure almost opens a gap with anti-crossing lines except for the pairs of the Weyl points (Fig. 1d, right). These topological electronic states including the Fermi arc are observed in the recent ARPES study, demonstrating that Co_3_Sn_2_S_2_ is a novel example of the magnetic WSM^20^.

The giant AH conductivity and AH angle are observed in Co_3_Sn_2_S_2_, both of which are categorized in the largest class among the known compounds^7^. Figure 1e, f shows the magnetic-field and temperature dependence of the Hall conductivity, respectively, for the bulk single crystal in the magnetic field parallel to the *c* axis. Below *T*_C_, which is discerned also as the anomaly in the temperature dependence of the resistivity (Fig. 1f), the AHE rapidly grows up and reaches 1300 Ω^−1^ cm^−1^ at the lowest temperature (Fig. 1e, f). The very similar transport properties are also confirmed for the thin film used in this study^21^ (Supplementary Fig. 2). Several recent studies suggested that the observed large AHE is attributed to the intense Berry curvature associated with the Weyl points and anti-crossing line structures^7,8^.

### MO effects

**Fig. 2:**
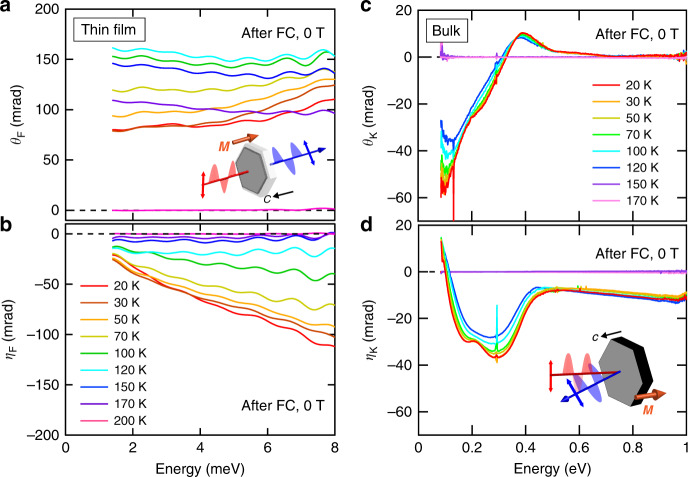
Terahertz Faraday and infrared Kerr rotations. **a** Terahertz Faraday rotation (*θ*_F_) spectra. Inset: the schematic illustration of the Faraday rotation measurement. **b** Faraday ellipticity (*η*_F_) spectra. **c** Infrared Kerr rotation (*θ*_K_) spectra. **d** Infrared Kerr ellipticity (*η*_K_) spectra. Inset: the schematic illustration of the Kerr rotation measurement. The measurements were done after the field cooling from 200 K.

To address the optical transition related to these topological electronic structures, we measured the terahertz Faraday rotation at 1–8 meV for the thin film and the infrared Kerr rotation at 0.08–1 eV for the bulk single crystal. Figure 2 shows the temperature dependence of the terahertz Faraday and infrared Kerr effects for the *c* plane at zero field after the field-cooling procedure; the field cooling is performed with applying the magnetic field along the *c* axis from 200 K (>*T*_C_), which stabilizes the single ferromagnetic domain state due to the easy-axis anisotropy along the *c* axis (Fig. 1e; see also Methods and Supplementary Fig. 3). The optical rotation owing to the terahertz Faraday and infrared Kerr effects grows up below *T*_C_. With decreasing the temperature, as a whole, the terahertz Faraday rotation *θ*_F_ is once enlarged and then reduced (Fig. 2a), whose low-energy limit (1.38 meV) shows the similar temperature dependence to the DC Hall angle (Supplementary Fig. 4). In the Faraday ellipticity *η*_F_ spectra, the negative slope as a function of the energy is observed, developing at low temperatures (Fig. 2b). Meanwhile, in the infrared region, where the interband optical transitions across the Weyl points are anticipated to occur, the magnitude of the Kerr rotation shows the monotonous increase while keeping the spectral characteristics unchanged. The Kerr rotation spectra *θ*_K_(*ω*) show the pronounced negative peak structure at 0.1 eV (Fig. 2c) and the Kerr ellipticity spectra *η*_K_(*ω*) show two negative peak structures at 0.15 and 0.3 eV (Fig. 2d). The rotation angles *θ*_F_(*ω*) and *θ*_K_(*ω*) reach 160 mrad (~9.4 deg) and 57 mrad (~3.2 deg) at maximum, respectively, both of which are remarkably large as discussed later.

### Longitudinal and Hall conductivity spectra

**Fig. 3:**
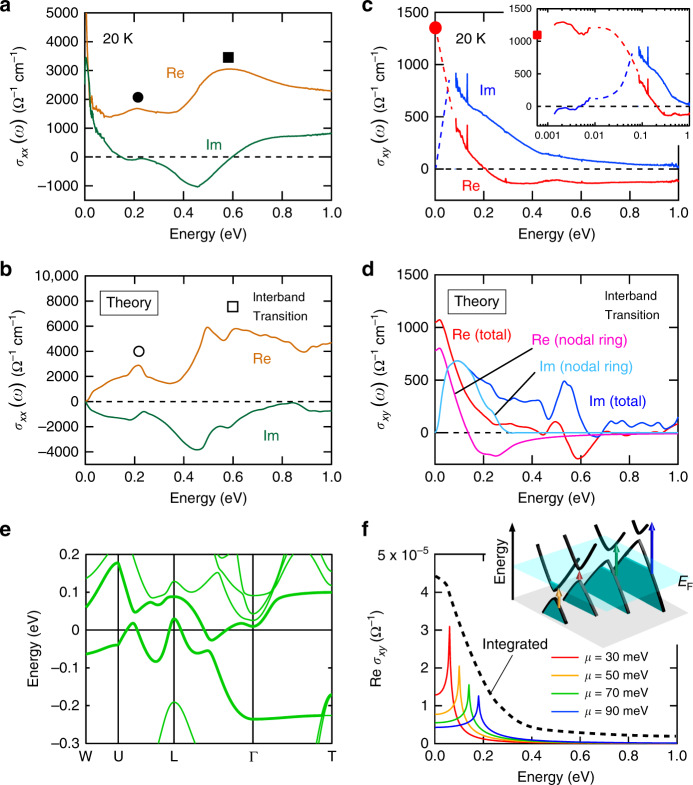
Longitudinal optical conductivity and optical Hall conductivity spectra obtained from the experiment and first-principles calculation. Experimental (**a**) longitudinal optical conductivity spectra *σ*_*xx*_(*ω*) and (**c**) optical Hall conductivity spectra *σ*_*xy*_(*ω*). The red circle at zero energy in **c** represents the DC value of the *σ*_*xy*_ of the bulk single crystal. The inset of **c** shows the optical Hall conductivity spectra including the terahertz spectra on the logarithmic energy scale. The red square at zero energy in the inset of **c** represents the DC value of the *σ*_*xy*_ of the thin film. The dashed lines in **c** for a photon energy region of 8–80 meV, where the MO measurements could not be done, represent the anticipated connections between the experimental *σ*_*xy*_(*ω*) values in THz/DC and infrared regions, assuming that no sharp resonance structure is present in this narrow energy window. Theoretical (**b**) longitudinal optical conductivity spectra *σ*_*xx*_(*ω*) and (**d**) Hall conductivity spectra *σ*_*xy*_(*ω*) (see Method). In **d**, the red and blue curves represent the *σ*_*xy*_(*ω*) spectra which take into account all band structures, while the pink and light blue ones show the *σ*_*xy*_(*ω*) arising from the topological bands indicated by bold lines in **e**. These theoretical calculations include only the interband transition, so that the response from the intraband transition or Drude response of conduction electrons is omitted. **e** The band structure obtained from the calculation considering the renormalization factor. The two bands indicated by bold curves compose the anti-crossing lines and Weyl points. **f** Optical Hall conductivity *σ*_*xy*_(*ω*) calculated for a single anti-crossing point in the two-dimensional electronic structure with mass gap *m* = 0.02 eV and chemical potential μ = 0.03 eV (red), 0.05 eV (orange), 0.07 eV (green), 0.09 eV (blue). The dotted line illustrates the schematic curve of the integrated sum of these resonances arising from the dispersive nodal ring. (Inset) Schematic illustration of the optical transitions on the cross-sections of the dispersive anti-crossing lines.

We show the longitudinal optical conductivity *σ*_*xx*_(*ω*) and the optical Hall conductivity *σ*_*xy*_(*ω*) for the comparison with the first-principles calculation as well as with the DC AHE (Fig. 3a–d). The *σ*_*xx*_(*ω*) spectra for the bulk single crystal were deduced through the Kramers–Kronig transformation of the reflectivity spectra at 20 K (Fig. 3a; see also Supplementary Fig. 5). The interband transitions including two peak structures at 0.2 (circle) and 0.6 eV (square) are observed in Re *σ*_*xx*_(*ω*) in addition to the strong zero-frequency peak due to the Drude response of conduction electrons below 0.02 eV (Fig. 3a). In fact, the Drude response is dominant for *σ*_*xx*_(*ω*) observed in the terahertz region (Supplementary Fig. 6). Apart from the Drude component, the overall spectral characteristics of the interband transitions are well reproduced by the theoretical calculation as shown in Fig. 3b; two broad peaks are identified at ~0.2 and 0.6 eV, as indicated by the open circle and square, respectively. The recent optical study argues that the lower-lying peak at 0.2 eV is mainly the transition among Co 3*d* orbital in nearly the same spin channel while the higher-lying peak at 0.6 eV is associated with the transition from Co 3*d t*_2g_ orbital to *e*_g_ orbital^31^. Some fine structures discerned in the calculated spectra (Fig. 3b) presumably result from the small damping of the optical transition assumed in the calculation. It is noted that, to consider the electron correlation effect, we introduced the renormalization factor of 1.52 and that the energy was divided by this factor for all theoretical spectra; this treatment is verified by the recent ARPES study, where the DFT calculation with the renormalization factor of 1.43 well reproduces the experimental band structure^20^.

The spectral responses in the MO Faraday/Kerr rotation are argued in a unified manner by the complex optical Hall conductivity spectra *σ*_*xy*_(*ω*). The *σ*_*xy*_(*ω*) spectra (Fig. 3c), which were deduced from the MO Faraday/Kerr rotation and *σ*_*xx*_(*ω*) spectra (see Method), are totally distinct from the longitudinal optical conductivity *σ*_*xx*_(*ω*) (Fig. 3a). Re *σ*_*xy*_(*ω*) increases below ~0.3 eV towards zero photon energy with the sign change at ~0.2 eV (red line, Fig. 3c) and approaches to the lowermost-energy value in the terahertz region (red line, inset of Fig. 3c) or equivalently to the DC value, indicating the monotonous rise of Re *σ*_*xy*_(*ω*) below 0.1 eV (red dotted line, Fig. 3c). On the other hand, Im *σ*_*xy*_(*ω*) gradually increases towards zero frequency below ~0.4 eV (blue line, Fig. 3c) and converges to zero in the terahertz region (blue line, inset of Fig. 3c) in accord with the causality constraint Im *σ*_*xy*_(*ω* = 0) = 0; therefore, the peak structure is inferred to position below 0.1 eV (blue dotted line, Fig. 3c). These spectral characteristics are consistent with the theoretical spectra that take the interband transition into account (Fig. 3d). Re *σ*_*xy*_(*ω*) gradually increases towards zero frequency below ~0.4 eV with forming the peak structure near zero frequency, while Im *σ*_*xy*_(*ω*) shows the broad peak structure around ~0.1 eV. Accordingly, the broad interband transition below 0.4 eV observed in both the experiment and theory is responsible for the DC AHE, evidencing the intrinsic origin. The possible extrinsic AHE is excluded because the spectral weight is distributed in a broader energy range than that of the Drude response with a scattering rate of ~3 meV in *σ*_*xx*_(*ω*) (Supplementary Fig. 6). The interband transition below 0.2 eV can be ascribed mainly to the optical transition across the Weyl points and anti-crossing lines in Co_3_Sn_2_S_2_; in fact, the low-energy spectra of total *σ*_*xy*_(*ω*) (red and blue) almost coincide with the band-resolved *σ*_*xy*_(*ω*) (pink and light blue) arising from interband transition between the two bands composing those topological electronic structures (bold curves in Fig. 3e), as shown in Fig. 3d. Thus, the optical Hall conductivity spectra demonstrate their vital role in producing the large AHE as well as the gigantic MO effect.

It should be emphasized that the broad spectral width of resonances in *σ*_*xy*_(*ω*) is naturally expected from the dispersive anti-crossing lines traversing the Fermi level in Co_3_Sn_2_S_2_ (Fig. 1c). As shown in Fig. 1a, a single anti-crossing point gives rise to the sharp resonance peak in the optical Hall conductivity. On the other hand, the cross-sections of the dispersive anti-crossing lines at different *k* points can be viewed as the two-dimensional two-band model (Fig. 1a) with different optical transition energies (Fig. 3f, inset). The integration of the optical response in *k* space thus results in the continuum resonance band in optical Hall conductivity *σ*_*xy*_(*ω*) (Fig. 3f). The anti-crossing point near the Fermi level has a larger resonance peak if assuming the constant mass gap. Therefore, the anti-crossing lines traversing the Fermi level results in the low-energy peak of *σ*_*xy*_(*ω*) with the large spectral weight, producing the large AHE at zero photon energy, i.e., DC AHE.

## Discussions

**Fig. 4:**
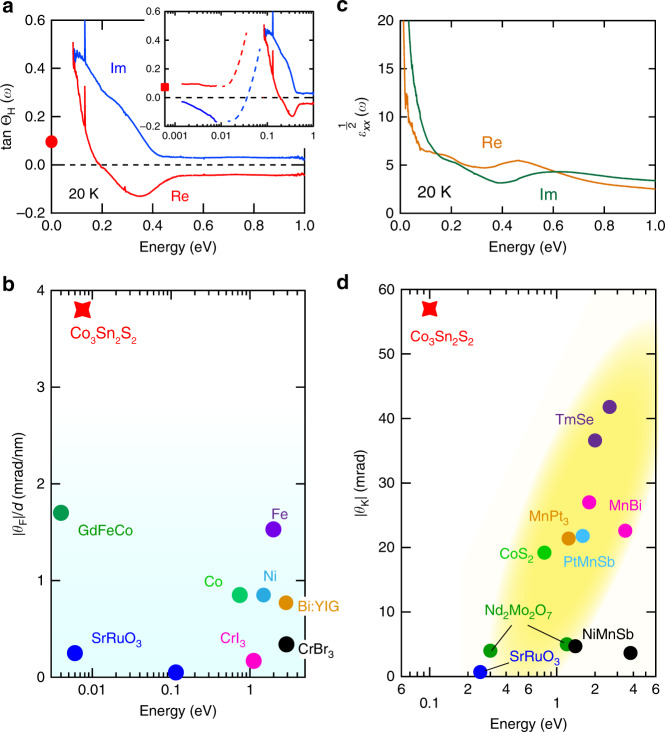
Large magneto-optical responses due to Berry curvatures. **a** Hall angle spectra, tanΘ_H_ = *σ*_*xy*_**/***σ*_*xx*_. The red circle at zero frequency represents the DC value of the Hall angle of the bulk single crystal. The inset shows the Hall angle spectra including the terahertz spectra on the logarithmic energy scale. The red square at zero frequency represents the DC value of the Hall angle of the thin film. **b** The peak magnitudes of the Faraday rotation angle divided by the sample thickness, *d*, for several ferromagnets. **c** Spectra of square root of the complex dielectric constant, *ε*_*xx*_^1/2^(*ω*). **d** The peak magnitudes of the Kerr rotation angle for several ferromagnetic metals.

As for the observed MO effect in this WSM compound, it is worth noting that both of the Faraday and Kerr rotations are proportional to the Hall angle, *σ*_*xy*_/*σ*_*xx*_ = tanΘ_H_(*ω*); *θ*_F_ + *iη*_F_ = *Z*_0_*σ*_*xy*_*d*/(1 + *n*_s_ + *Z*_0_*σ*_*xx*_*d*) ~ tanΘ_H_(*ω*) for thin film and *θ*_K_ + *iη*_K_ = −tanΘ_H_(*ω*)/*ε*_*xx*_^1/2^ for bulk surface, where *n*_s_, *Z*_0_, and *ε*_*xx*_ are the refractive index of the substrate, vacuum impedance, and the dielectric constant, respectively (See also Methods). In the present case, the real part of tanΘ_H_(*ω*) shows the maximum as large as 0.5 at ~0.1 eV (Fig. 4a) due to the optical transitions on the anti-crossing lines and Weyl points near the Fermi level.

The Faraday rotation observed at zero field, which exceeds 160 mrad (3.8 mrad/nm), is larger than that of the conventional ferromagnets represented by the archetypal MO material Bi:YIG with 0.77 mrad/nm^25,26,32–35^ (Fig. 4b). We note that the difference in the refractive indices for right and left circularly polarized light Δ*n* and the figure of merit defined by *ω*Δ*nd*_p_/2*c*, where *d*_p_ is the penetration depth, are more appropriate for the quantitative discussion of the Faraday rotation, because the magnitude of the Faraday rotation for thin films depends on the configuration of film; the |Δ*n*| and |*ω*Δ*nd*_p_/2*c*| of the present material take large values ~10.6 and 451 mrad, respectively at 7.5 meV (Supplementary Note 2 and Supplementary Table 1). Moreover, the large infrared Kerr rotation has never been reported for the (ferro)magnetic metals because it should be necessarily suppressed by the divergence of *ε*_*xx*_^1/2^ due to the Drude response of the conduction electron towards zero frequency (Fig. 4c) as suggested by the relation that *θ*_K_ + *iη*_K_ = −tanΘ_H_(*ω*)/*ε*_*xx*_^1/2^. In fact, the peak magnitudes of the Kerr rotation angle for several ferromagnetic metals tend to be small in low-energy regions^23,25,26,36–40^ (Fig. 4d). In Co_3_Sn_2_S_2_, by contrast, the Kerr rotation is enhanced even in the far-infrared region beyond this tendency owing to the large Hall angle originating from the topological electronic structure. These strong MO responses exemplify the novel optical property inherent to the magnetic WSM. It should be noted that these topological MO responses are in contrast with the usual plasma-edge enhancement, in which the reduction of *ε*_*xx*_ around the plasma edge increases the MO signal from near-infrared to visible regions^39^.

In conclusion, we have studied the MO responses in magnetic WSM Co_3_Sn_2_S_2_. Our work reveals two intriguing aspects of the WSM, the optical Hall conductivity associated with the AHE and the enhanced MO effects, both of which originate from the Berry curvature of the topological electronic structures. The comprehensive study based on the MO spectroscopy and first-principles calculation clearly establishes the low-energy electron dynamics arising from the topological electronic structures, which is the source of the large intrinsic AHE. One other important aspect is that the optical transition related to the Weyl point/anti-crossing line produces the enhanced MO effects. This mechanism is essentially distinct from the conventional plasma-edge enhancement and applies to many other topological materials including even nonmagnetic Dirac or WSMs in general^11–18^, promising future optical/electronic applications.

## Methods

### Single crystal growth

Single crystalline samples of Co_3_Sn_2_S_2_ were prepared by Bridgman methods. Co, Sn, and S were mixed in a stoichiometric ratio and then sealed in a quartz tube. Afterwards, it was heated to 1323 K and cooled down to 973 K with a rate of 4 mm per hour. The single phase with a shandite-type structure was confirmed by using the powder x-ray analysis.

### Thin film fabrication

The 42-nm-thick *c*-axis-oriented Co_3_Sn_2_S_2_ film capped with a 50-nm-thick insulating SiO_2_ layer was grown by radio-frequency magnetron sputtering on Al_2_O_3_ (0001) substrates. The crystal structure and composition were confirmed by x-ray diffraction and energy-dispersive X-ray spectroscopy, respectively.

### Transport measurement

The magneto-resistivity and Hall resistivity were measured by using Physical Property Measurement System (Quantum Design).

### MO Kerr effect measurement in infrared regions

MO Kerr rotation spectra were measured with the use of a photoelastic modulator^41^. The detection of the synchronous signal of the reflected light with the fundamental and second harmonic of the modulation frequency enables us to measure the Kerr rotation *θ*_K_ and ellipticity *η*_K_, respectively. For the measurement, we performed the field cooling in the magnetic field ~70 mT using a permanent magnet from 200 K, resulting in the single magnetic domain state. During the measurement, the permanent magnet was removed. To deduce the Kerr rotation spectra, we anti-symmetrized the spectra for the positive and negative magnetizations.

### Optical (longitudinal) conductivity *σ*_*xx*_(*ω*) and Hall conductivity *σ*_*xy*_(*ω*) spectra in infrared regions

The optical conductivity spectra were deduced through the Kramers–Kronig transformation of the reflectivity spectra from 0.01 to 5 eV. For the extrapolation of the reflectivity data, we used the Hagen–Rubens relation below the lowest energy measured and assumed that the reflectivity is proportional to *ω*^−4^ above the highest energy. The optical Hall conductivity spectra were calculated by using the following formula; *σ*_*xy*_(*ω*) = −*σ*_*xx*_(*ω*)*ε*_*xx*_^1/2^(*ω*)(*θ*_K_(*ω*) + *iη*_K_(*ω*)).

### Terahertz time-domain spectroscopy (THz-TDS)

In the THz-TDS, laser pulses with a duration of 100 fs from a mode-locked Ti:sapphire laser were split into two paths to generate and detect THz pulses using the photoconductive antenna. Transmittance spectra were obtained by measuring the transmission of both the sample and substrate. We used the following standard formula to obtain the complex conductivity *σ*_*xx*_(*ω*) = *σ*_1_(*ω*) + *iσ*_2_(*ω*) of the thin film;

$$\begin{array}{*{20}{c}} {t\left( \omega \right) = \frac{{1 + n_s}}{{1 + n_s + Z_0d{\upsigma}_{xx}\left( \omega \right)}},} \end{array}$$

where *t*(*ω*), *d*, *Z*_0_, and *n*_s_ are the complex transmittance, the thickness of the film, the vacuum impedance (377 Ω), and the refractive index of the sapphire substrate, respectively. As the reference, we used the bare sapphire substrate to deduce the optical conductivity of the Co_3_Sn_2_S_2_ thin film. We note that the terahertz conductivity of SiO_2_ cap layer is negligibly small compared with that of the Co_3_Sn_2_S_2_ thin film and therefore the SiO_2_ layer hardly contributes to the terahertz spectra (Supplementary Fig. 7).

### Terahertz Faraday rotation measurements

The rotatory component of the transmitted THz pulses, *E*_*y*_(*t*), which is the electric field perpendicular to the incident electric field, *E*_*x*_(*t*), was measured in the crossed-Nicole configuration by using wire-grid polarizers. To eliminate the background signal, we calculated the polarization rotation *E*_*y*_(*t*) by anti-symmetrizing the rotatory components under positive and negative magnetic fields. The Fourier transformation of the THz pulses *E*_*x*_(*t*) and *E*_*y*_(*t*) gives the complex Faraday rotation spectra *θ*_F_(*ω*) + *iη*_F_(*ω*) = tan^−1^(*E*_*y*_(*ω*)/*E*_*x*_(*ω*)). For the anti-symmetrization, we performed the field cooling in the magnetic field of ±1 T from 200 K, resulting in the single magnetic domain state^21^. During the terahertz MO measurement, the magnetic field was absent.

### Terahertz Hall conductivity

By using the longitudinal conductivity spectra *σ*_*xx*_(*ω*) and Faraday rotation spectra *θ*_F_(*ω*) + *iη*_F_(*ω*), the Hall conductivity spectra were calculated from the following formula; $\sigma _{xy}\left( \omega \right) = (\theta _{\mathrm{F}} + i\eta _{\mathrm{F}})\frac{{1 + n_s + Z_0d\sigma _{xx}\left( \omega \right)}}{{Z_0d}}$.

### DFT calculation

The electronic structure of Co_3_Sn_2_S_2_ was calculated by using the *OpenMX* code^42^, where the exchange-correlation functional within the local spin density approximation^43^ and norm-conserving pseudopotentials^44^ were employed. The spin–orbit coupling was included by using total angular momentum dependent peudopotentials^45^. The wave functions were expanded by a linear combination of multiple pseudoatomic orbitals^46^. A set of pseudoatomic orbital basis was specified as Co6.0-*s*3*p*3*d*3, Sn7.0-*s*4*p*3*d*1, where the number after each element stands for the radial cutoff in the unit of Bohr and the integer after *s*, *p*, and *d* indicates the radial multiplicity of each angular momentum component. The cutoff-energy for charge density of 350.0 Ry and a *k*-point mesh of 31 × 31 × 31 were used. The lattice constant of Co_3_Sn_2_S_2_ was set to *a* = 5.36 and *c* = 13.17 Å^7^. The magnetic moment is evaluated as 0.9 μ_B_/f.u. in this calculation.

### Wannier representation and optical conductivities

From the Bloch states obtained in the DFT calculation described above, a Wannier basis set was constructed by using the *Wannier90* code^47^. The basis was composed of (*s*, *p*, *d*)-character orbitals localized at each Co and Sn site and (*s*, *p*)-character ones at S site, which are 106 orbitals/f.u. of Co_3_Sn_2_S_2_ in total if including the spin multiplicity. These sets were extracted from 194 bands in the space spanned by the original Bloch bands at the energy range from −20 to +50 eV.

The optical conductivity and optical Hall conductivity were calculated by using Kubo-Greenwood formula given by,

$$\sigma _{\alpha \beta }\left( {\hbar \omega } \right) = \frac{{ie^2\hbar }}{{N_k{\mathrm{\Omega }}_c}}\mathop {\sum }\limits_{{\boldsymbol{k}},n,m} \frac{{f_{m,{\boldsymbol{k}}} - f_{n,{\boldsymbol{k}}}}}{{\varepsilon _{m,{\boldsymbol{k}}} - \varepsilon _{n,{\boldsymbol{k}}}}}\frac{{\langle \psi _{n,{\boldsymbol{k}}}{\mathrm{|}}v_\alpha {\mathrm{|}}\psi _{m,{\boldsymbol{k}}}\rangle\langle\psi _{m,{\boldsymbol{k}}}{\mathrm{|}}v_\beta {\mathrm{|}}\psi _{n,{\boldsymbol{k}}}\rangle}}{{\varepsilon _{m,{\boldsymbol{k}}} - \varepsilon _{n,{\boldsymbol{k}}} - \left( {\hbar \omega + i\eta } \right)}},$$

where *e*, $\hbar$, Ω_c_, *N*_*k*_, *η*, $f_{n,{\boldsymbol{k}}}$ are the elementary charge with negative sign, reduced Planck constant, cell volume, number of *k*-point, smearing parameter, and the Fermi–Dirac distribution function with the band index *n* and the wave vector ***k***, respectively. The $\sigma _{\alpha \beta }\left( {\hbar \omega } \right)$ was calculated using the Wannier-interpolated band structure with a 100 × 100 × 100 *k*-point grid and *η* = 20 meV. To consider the electron correlation effect, we introduced the renormalization factor of 1.52 phenomenologically and the energy was divided by this factor.

## Supplementary information

Supplementary Information

## Data Availability

The data that support the plots of this study are available from the corresponding author upon reasonable request.
