# Supplementary material for: Giant magneto-optical responses in magnetic Weyl semimetal Co3Sn2S2
Source: Nat Commun. 2020 Sep 15;11:4619. doi: 10.1038/s41467-020-18470-0 (PMC7492236; doi:10.1038/s41467-020-18470-0)
Supplement: Supplementary file 1 — Supplementary Information [file 41467_2020_18470_MOESM1_ESM.pdf]

Supplementary Information for

Giant magneto-optical responses

in magnetic Weyl semimetal $Co_3Sn_2S_2$

Y. Okamura[1*], S. Minami[2,3], Y. Kato[1], Y. Fujishiro[1], Y. Kaneko[3], J. Ikeda[4], J. Muramoto[1], R. Kaneko[1], K. Ueda[1], V. Kocsis[3], N. Kanazawa[1], Y. Taguchi[3], T. Koretsune[5], K. Fujiwara[4], A. Tsukazaki[4], R. Arita[1,3], Y. Tokura[1,3,6] and Y. Takahashi[1,3*]

[1]*Department of Applied Physics and Quantum Phase Electronics Center, University of Tokyo, Tokyo 113-8656, Japan*

[2]*Nanomaterials Research Institute, Kanazawa University, Ishikawa 920-1192, Japan*

[3]*RIKEN Center for Emergent Matter Science (CEMS), Wako 351-0198, Japan*

[4]*Institute for Materials Research, Tohoku University, Sendai 980-8577, Japan*

[5]*Deparment of Physics, Tohoku University, Sendai 980-8578, Japan*

[6]*Tokyo College, University of Tokyo, Tokyo 113-8656, Japan*

[*]To whom correspondence should be addressed (okamura@ap.t.u-tokyo.ac.jp, youtarou-takahashi@ap.t.u-tokyo.ac.jp)

Supplementary Note 1: Hall conductivity spectra for the single anti-crossing point.

The single anti-crossing point in the two-dimensional electronic structure was employed as the simplest model for the anomalous Hall effect (Fig. 1a in the main text), which is expressed by the following Hamiltonian $H(k)$,

$$H(k) = -\mu\sigma_0 + \sum_{i=x,y,z} h_i(k)\sigma_i,$$

where σ_0 and σ_i (i=x, y, z) are the identity and Pauli matrices, respectively, and μ is the chemical potential. Assuming that the band dispersion is two-dimensional and (h_x, h_y, h_z) = (k_x, k_y, m), the corresponding band dispersion has the level splitting of $2|m|$ at the Weyl point, which is schematically illustrated in the inset of Fig. 1a in the main text.

We calculated the optical Hall conductivity $\sigma_{xy}(\omega)$ with use of the general expression given by the Kubo formula;

$$\sigma_{xy}(\omega) = i\sum_{n,m}\frac{f(\varepsilon_m) - f(\varepsilon_n)}{\varepsilon_m - \varepsilon_n}\frac{\langle m|J_y|n\rangle\langle n|J_x|m\rangle}{\omega + i\gamma + \varepsilon_m - \varepsilon_n}$$

where the $J_{x(y)}$ is the current operator given by $\frac{\hbar}{e}\sum_k c^\dagger(k)\frac{\partial H(k)}{\partial k_{x(y)}}c(k)$, $f(\varepsilon_n)$ is the Fermi distribution function, ε_n and $|n\rangle$ are the energy and the Bloch wave function of the n-th band, respectively, and γ is the damping constant. The energy-dependent Hall conductivity $\sigma_{xy}(\omega)$ is thus given by;

$$\sigma_{xy}(\omega) = \frac{e^2}{2h}\frac{m}{\hbar\omega+i\gamma}\ln\left|\frac{-\hbar\omega-i\gamma+2\mu}{\hbar\omega+i\gamma+2\mu}\right|.$$

For the calculations shown in the main text, we assumed $\gamma = 1$ meV.

Supplementary Note 2: Quantitative discussion of the Faraday rotations.

The Faraday rotation for the free-standing bulk crystal is given by,

$$\theta_F + i\eta_F = -\frac{i\omega d}{2c}\frac{\varepsilon_{xy}}{\sqrt{\varepsilon_{xx}}} = -\frac{\omega d}{2c}\Delta n,$$

where c and d are the speed of light and thickness of the sample, respectively. Δn represents the difference in the refractive indices for the right and left circularly polarized light. Therefore, the θ_F/d is a good measure to evaluate the magnitude of the Faraday rotation quantitatively for the bulk case, i.e. $\omega d/c >> 1$. However, in case of the thin film on top of the substrate, the terahertz Faraday rotation is given by, $\theta_F + i\eta_F = Z_0\sigma_{xy}d/(1+n_s+Z_0\sigma_{xx}d)$, as described in Method section; thus, the θ_F is the nonlinear function of d and does not necessarily represent the intrinsic material parameters. Therefore, to discuss the magnitude of the Faraday rotation in a unified manner, the Δn and the figure of merit defined by $\omega\Delta n d_p/2c$, where d_p is the penetration depth, are more appropriate. Here, for the $Co_3Sn_2S_2$ thin film, $|\Delta n|$ and $|\omega\Delta n d_p/2c|$ are estimated to be 10.6 and 451 mrad, respectively at 7.5 meV, both of which are much larger than those of YIG at 4.16 eV [S1,2] (Supplementary Table 1). We also calculate the θ_F/d for the free-standing film ($\theta_F^{calc.}/d$); it decreases from the experimentally observed $\theta_F^{obs.}/d$, but is still larger than that of YIG.

	Energy	$\lvert\theta_F^{obs.}/d\rvert$	$\lvert\Delta n\rvert$	$\lvert\omega\Delta n d_p/2c\rvert$	$\lvert\theta_F^{calc.}/d\rvert$	Ref.
$Co_3Sn_2S_2$	7.5 meV	3.8 mrad/nm	10.6	451 mrad	0.202 mrad/nm	This work
YIG	4.16 eV	0.175 mrad/nm	0.0144	4.97 mrad	0.151 mrad/nm	[S1,2]
$SrRuO_3$	6 meV	0.25 mrad/nm	0.469	8.05 mrad	0.0071 mrad/nm	[S3]
$SrRuO_3$	0.115 eV	0.05 mrad/nm	0.0996	27.0 mrad	0.029 mrad/nm	[S4]

Supplementary Table 1| Quantitative comparison of Faraday rotation. The $\theta_F^{obs.}/d$ represents the experimentally observed Faraday rotation $\theta_F^{obs.}$ divided by the film thickness d. We also calculate the difference in the refractive indices for right and left circularly polarized light Δn and the figure of merit for the Faraday rotation, $\omega\Delta n d_p/2c$, where d_p is the penetration depth. The θ_F/d is the rotation angle the free-standing film ($\theta_F^{calc.}/d$) obtained from the calculation.

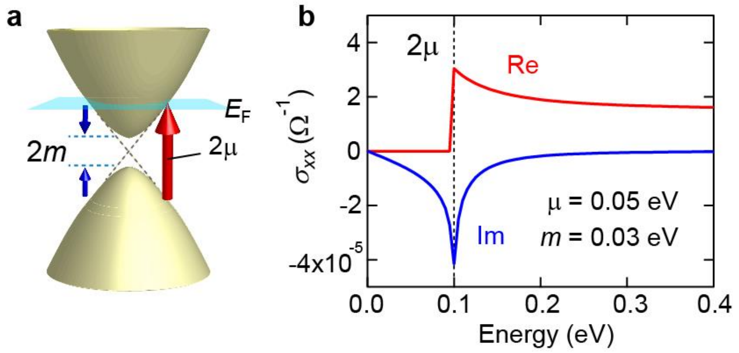

Supplementary Figure 1| Optical conductivity spectra $\sigma_{xx}(\omega)$ calculated for the single anti-crossing point. a The schematic illustration of the single anti-crossing point. **b** The optical conductivity spectra except for the Drude components calculated for a single anti-crossing point in the two-dimensional electronic structure with mass gap m = 0.03 eV and chemical potential μ = 0.05 eV (For the detail of the calculation, see Ref[S5]). Re $\sigma_{xy}(\omega)$ and Im $\sigma_{xx}(\omega)$ show the sharp resonance peaks, while Im $\sigma_{xy}(\omega)$ and Re $\sigma_{xx}(\omega)$ have the step-function like structure at the interband transition energy (2μ) (Fig. 1a).

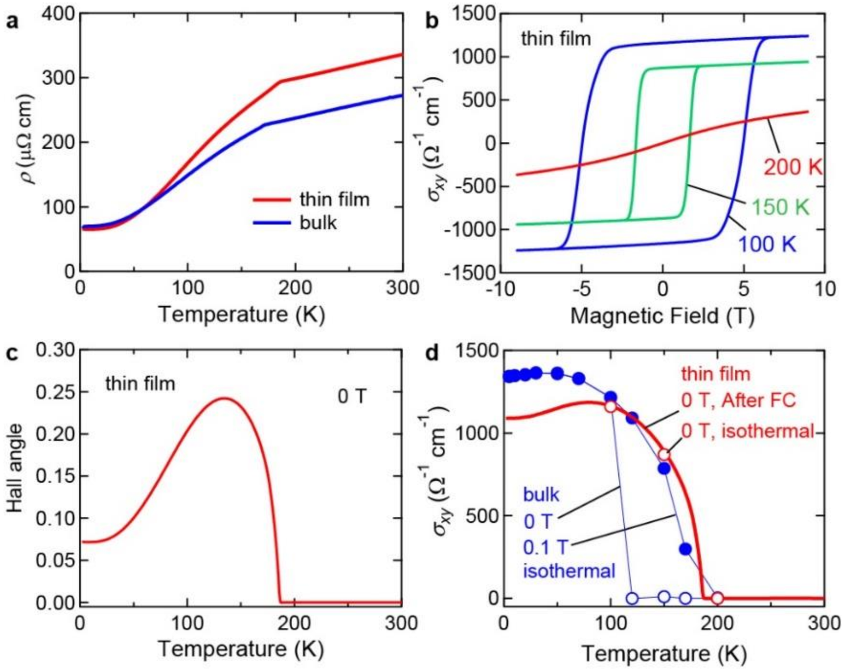

Supplementary Figure 2| Transport properties of $Co_3Sn_2S_2$ thin film and comparison with the bulk. a Temperature dependence of resistivity for thin film (red line) and for the bulk single crystal (blue line). **b** Magnetic-field dependence of the Hall conductivity for thin film. **c** Temperature dependence of the Hall angle measured at 0 T after the field cooling in the magnetic field of 1 T. **d** Temperature dependence of the Hall conductivity for thin film measured at 0 T after the field cooling in the magnetic field of 1 T (red line). The red open circles are taken from the isothermal measurement in **b**. The magnitudes are in good agreement for both measurement procedures, indicating the stable single ferromagnetic domain state in zero field. The Hall conductivity at 0 T and 0.1 T for the bulk single crystal are also indicated by the blue open and filled circles, respectively. The zero-field Hall conductivity disappears above 120 K because the single domain state is unstable in zero field near T_C. Overall characteristics of transport properties including the magnitude are comparable between the bulk and thin film. We note that the T_C of the thin film (~ 184 K) is higher than that of the bulk (~ 172 K), which

is possibly due to slight composition discrepancy because the T_C tends to scatter in sample to sample in this system. One other possible cause is the strain effect. Since the reduction of the T_C by applying isotropic pressure has been reported for the bulk system[S6], the uniaxial pressure effect for the thin film may play a role.

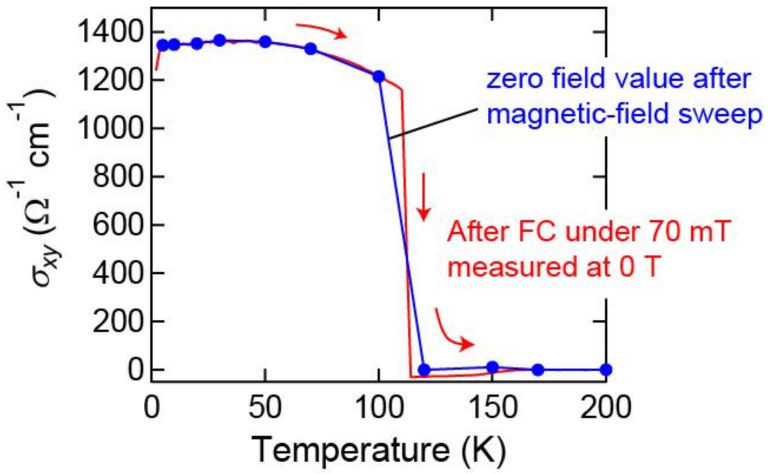

Supplementary Figure 3| Confirmation of the single domain state after the field cooling (FC) for the bulk single crystal. The red curve shows the zero-field Hall conductivity measured after the FC in 70 mT. The blue curve shows the Hall conductivity measured after the magnetic field sweep at each temperature. These Hall conductivities coincide with each other, indicating the stable single domain state after the FC below ~ 120 K.

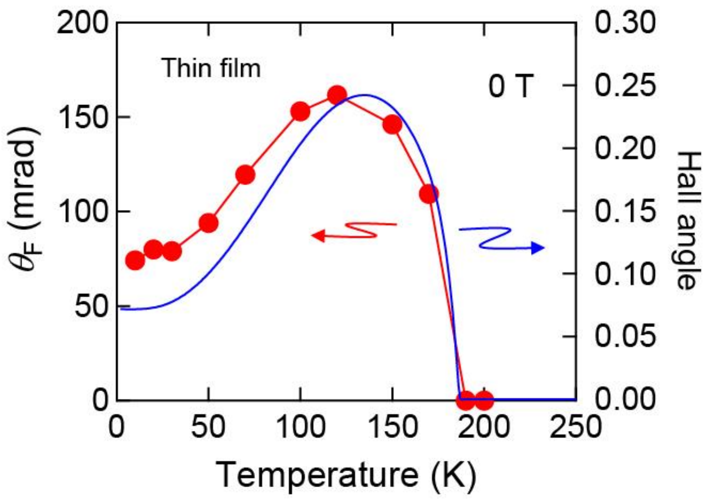

Supplementary Figure 4| Temperature dependence of the terahertz Faraday rotation and DC Hall angle for the thin film. Temperature dependence of the terahertz Faraday rotation at 1.38 meV (red circles) and DC Hall angle (blue line).

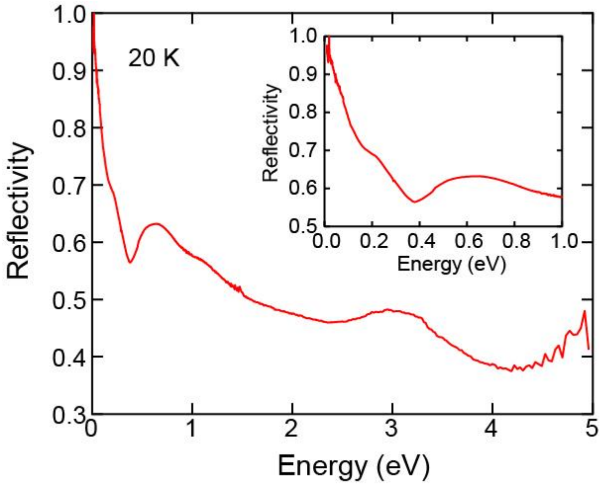

Supplementary Figure 5| Reflectivity spectra of $Co_3Sn_2S_2$ single crystal at 20 K. The reflectivity spectra from 0.01 to 5 eV. The inset shows the magnified view of the reflectivity spectra below 1 eV.

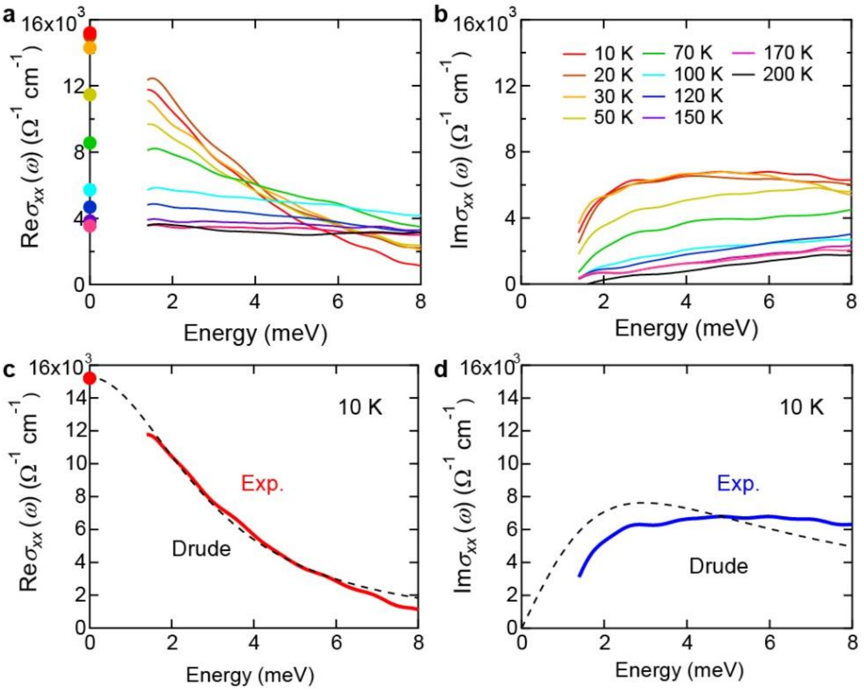

Supplementary Figure 6| Terahertz conductivity spectra of $Co_3Sn_2S_2$ thin film. a,b Terahertz conductivity spectra. Real part (**a**) and imaginary part (**b**). **c,d** Fitting of the terahertz spectra at 10 K with using the Drude model; $\sigma(\omega) = \frac{\sigma_0}{1-i\omega\tau}$. The experimental spectra (red and blue lines) are consistent with the Drude response with σ_0 = 15256.8 Ω^{-1} cm^{-1} and $1/\tau$ = 2.96 meV (dotted lines). The deviation from the fitting with the Drude model is discerned in **d**, which might suggest the presence of multiple Drude components. Nevertheless, this fitting can provide the evaluation of average scattering time of this thin film sample.

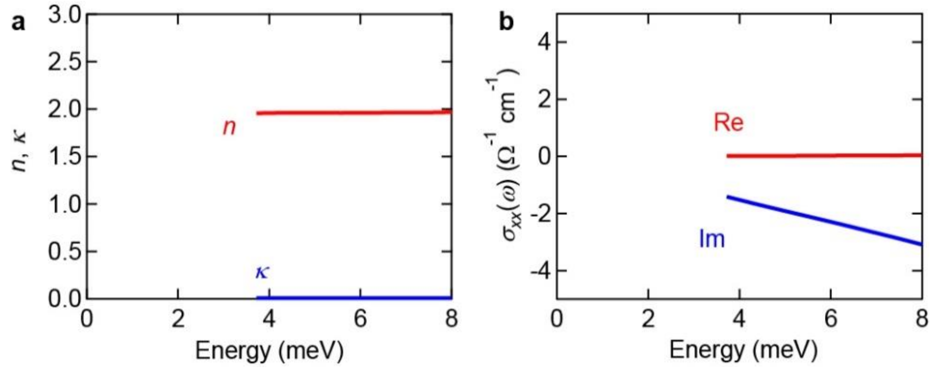

Supplementary Figure 7| Terahertz conductivity spectra of SiO_2. a The refractive index *n* and extinction coefficient *κ* in terahertz region reproduced from Ref[S7]. **b** The terahertz conductivity calculated from *n* and *κ* in **a**. The terahertz conductivity of SiO_2 is negligibly small compared with that of the $Co_3Sn_2S_2$ film, and therefore the possible modification of the terahertz spectra owing to the SiO_2 capping layer can be neglected within the accuracy of our measurement.

Supplementary Reference

S1. Kahn, F. J., Pershan, P. S. and Remeika, J. P. Ultraviolet magneto-optical properties of single-crystal orthoferrites, garnets, and other ferroic oxide compounds. *Phys. Rev.* **186,** 891 (1969).

S2. MacDonald, R. E. *et al*. Magneto-optical properties of garnet films. *J. Appl. Phys.* **38,** 4101 (1967).

S3. Shimano, R. *et al*. Terahertz Faraday rotation induced by an anomalous Hall effect in the itinerant ferromagnet $SrRuO_3$. *Europhys. Lett.* **95,** 17002 (2011).

S4. Kim, M.-H. *et al*. Determination of the infrared complex magnetoconductivity tensor in itinerant ferromagnets from Faraday and Kerr measurements. *Phys. Rev. B* **75,** 214416 (2007).

S5. Tse, W-K. & MacDonald, A. H. Giant magneto-optical Kerr and universal Faraday effect in thin-film topological insulators. *Phys. Rev. Lett.* **105,** 057401 (2010).

S6. Liu, Z. Y. *et al*. Pressure effect on the anomalous Hall effect of ferromagnetic Weyl semimetal $Co_3Sn_2S_2$. *Phys. Rev. Materials* **4,** 044203 (2020).

S7. Hosako, I. Optical thin film technology used in the terahertz frequency. *J. Natl. Inst. of Inf. Commun. Tech.* **51,** 87 (2004).
